# Two-Level Model for Detecting Substation Defects from Infrared Images

**DOI:** 10.3390/s22186861

**Published:** 2022-09-10

**Authors:** Bing Li, Tian Wang, Zhedong Hu, Chao Yuan, Yongjie Zhai

**Affiliations:** Department of Automation, North China Electric Power University, Baoding 071003, China

**Keywords:** infrared image, substation equipment, defect detection, superpixel segmentation, temperature probability density

## Abstract

Training a deep convolutional neural network (DCNN) to detect defects in substation equipment often requires many defect datasets. However, this dataset is not easily acquired, and the complex background of the infrared images makes defect detection even more difficult. To alleviate this issue, this article presents a two-level defect detection model (TDDM). First, to extract the target equipment in the image, an instance segmentation module is constructed by training from the instance segmentation dataset. Then, the target equipment is segmented by the superpixel segmentation algorithm into superpixels according to obtain more details information. Next, a temperature probability density distribution is constructed with the superpixels, and the defect determination strategy is used to recognize the defect. Finally, experiments verify the effectiveness of the TDDM according to the defect detection dataset.

## 1. Introduction

Substation equipment is an essential part of the power system [1]. Once defects exist in operating equipment, an abnormal temperature usually occurs at the defective parts, triggering thermal failures that can lead to local equipment burnout or even more severe electric power accidents [2]. Therefore, timely and accurate detection of defects in substation equipment is of great significance to the safety and stability of a power system.

Many methods have been studied for defects detection in substation equipment, including dielectric loss measurement [3], UHF (ultra-high frequency) method [4], FDR (frequency domain reflectometry) method [5], and infrared image-based methods [6,7]. The dielectric loss measurement requires off-line preventive testing, which will delay the operation of substation equipment. The complexity of the UHF method makes directly locating defective regions difficult. The FDR method is sensitive only to defects caused by moisture. Early infrared image-based methods for detecting thermal defects in substation equipment require manual intervention, which is time-consuming and costly. However, with the development of smart grids and the successful application of substation inspection robots, a large number of on-site infrared images needed to be inspected urgently. Intelligent defect detection methods have emerged based on computer vision.

Due to the redundant background and the densely packed targets, applying automatic intelligent defect detection methods directly is difficult. Thus, extracting the target equipment in the complex infrared images is required first. Early researchers studied the methods using traditional digital image processing techniques, including threshold-based, region-based, and edge-based methods. Threshold-based methods separate the foreground from the background of an image by selecting a suitable threshold [8], which is simple and efficient but susceptible to noise interference, causing poor robustness. A typical region-based method is the watershed algorithm [9]. It uses the local minima of the image gradient to form a specific region to segment different image parts. However, it is sensitive to the color changes in the object’s surface, giving rise to over-segmentation. Edge-based methods extract edge features from the image by edge detection operators such as the Sobel operator [10] and Canny operator [11] to realize the segmentation of an image. Nevertheless, it cannot guarantee the existence of closed, continuous edge regions, and it lacks robustness to noise interference. The recent rapid development of deep learning and imaging technologies has brought innovative ideas for extracted methods from infrared images of substation equipment. Instance segmentation is a classic task in the field of computer vision, which can perform object extraction excellently in images. This task, not only locates and classifies all instances but also segments each instance from the images [12]. Many applications benefit from accurate instance segmentation, including electrical systems [13,14], autonomous driving [15], robotics [16,17], and intelligent transportation systems [18]. Consequently, instance segmentation has become an active research topic in the industry, which benefits its powerful ability of object extraction. Xiong et al. [19] proposed a method based on Mask R-CNN and Bayesian context network to recognize power equipment, which is considered the relationship between objects in a complex background. Ling et al. [20] presented a novel deep learning framework to locate the broken insulators, which is address the problem of low signal-noise-ratio (SNR) setting. To detect the transmission line, a transmission line detection (TLD) algorithm is proposed [21], which is a multitask deep neural network with branched outputs. The deep learning-based methods show excellent performance to extract the target object.

In the stage of defect detection, some promising methods for detecting defects are feature extraction and convolutional neural networks. The key to feature extraction-based approaches is acquiring target ontology features and using classifiers to recognize the extracted features [22,23]. However, the effectiveness of feature extraction and the selection of classifiers are great dependence on personal experience. Convolutional neural networks focus on detecting target defects through an object detection model [24,25]. Li et al. [26] proposed a method of insulator defect location, which is cascades detection and segmentation networks from two levels. In view of the characteristics of insulator defects, Wang et al. [27] presented an improved network to detect the defect of aerial insulator photos. The above method achieved excellent results in defect detection, but requires numerous defective insulator images to train the DCNN. In reality, the infrared images of defective substation equipment are difficult to acquire, and the performance of DCNN is difficult to guarantee. Implementing defect detection of substation equipment in infrared images is still challenging. In an infrared image, the different parts of the target corresponding to different heat generation characteristics. Thus, the temperature feature of the target is used to estimate temperature probability density distribution, which is used to identify defects by the presented strategy. The proposed defect detection part is an unsupervised learning method and is not limited by the dataset. Before that, the superpixel processing is used to provide more details, those details offer more information for defect detection. Meanwhile, it reduces the complexity and time spent on the model.

This study proposes the TDDM for defect detection in electric power substations, which is used in infrared images of substation equipment, e.g., insulator, current transformer, lightning arrester, bushing and voltage transformer. The main contributions of this paper are as follows.

(1) Inspecting the substation equipment from the infrared images with the redundant background and the densely packed targets directly is difficult. The proposed TDDM extracted the target firstly, and then, defect analysis is conducted on a single instance, which is converted to a two-level detection problem.
(2) Superpixel segmentation is conducted on the extracted target equipment to merge adjacent pixels with similar characteristics. The process is used to provide more details and reduce the complexity of the subsequent detection determination.
(3) Based on a Gaussian kernel function, the temperature probability density distribution of the target equipment is constructed, which is used in a defect determination strategy to find the defective areas in infrared images of the target substation equipment.
(4) The experimental results show that the proposed model accurately detects defects in substation equipment in infrared images.

The remainder of this paper is organized as follows. In Section 2, a novel model for detecting these defects in infrared images is provided, including instance segmentation, superpixel segmentation, and defect determination. Section 3 verifies the performance of the proposed model and discusses the influences of superpixel parameters on the results. Section 4 concludes this work.

## 2. Procedure for the Proposed Model

**Figure 1:**
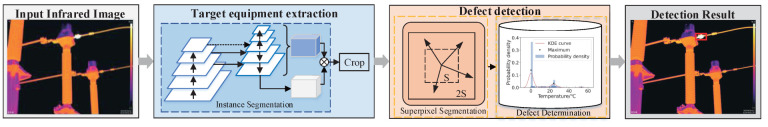
Flowchart of TDDM.

The model proposed is designed for automatically detecting defects of substation equipment in infrared images.The model transforms defect detection into a two-level detection problem. First, an instance segmentation algorithm directly extracts the target equipment from infrared images with complex backgrounds. After that, a superpixel segmentation algorithm merges similar characteristics and captures the details of the target equipment. Finally, the defect position is determined. Figure 1 is a flowchart of the proposed TDDM procedure.

### 2.1. Instance Segmentation

To detect substation equipment in infrared images, we must first extract the target equipment from the image. Instance segmentation is a basic task of DCNN, which is to extract the target from a complex background and distinguish different instances in the image’s foreground [28]. There are three commonly used instance segmentation methods: top-down detection-based methods, bottom-up semantic segmentation-based methods and direct instance segmentation at the pixel level. Top-down detection-based methods perform instance segmentation in a bounding box, such as the Mask R-CNN [29], Mask Scoring R-CNN [30], and YOLACT [31] networks. In bottom-up semantic segmentation-based methods, the pixels are labeled for prediction and clustered [32,33]. The SOLO algorithm [34] performs end-to-end optimization of instance segmentation by mask labeling, which directly segments instances at the pixel level.

This study extracted target equipment images using YOLACT. Its backbone is the feature extraction part used to obtain different resolution feature maps $C_{i}\left(i=2,3,4,5\right)$ from the input infrared image. The description of specific backbone configuration parameters as shown in Table 1. To obtain the multiscale features, $C_{i}\left(i=2,3,4,5\right)$ are fused by the horizontal connection with the feature pyramid. Then multiscale features $P_{j}\left(j=3,4,5,6,7\right)$ are connected to prediction heads for multiscale prediction of objects. There are two branches after the feature pyramid. The one branch predicts the object category, the bounding box, and the mask coefficients; the higher score bounding box is obtained through non-maximum suppression (NMS) [35]. The other branch is a fully convolutional network called protonet, which generates a series of prototype masks based on the feature map $P_{3}$. Finally, the prototype masks obtained from protonet are linearly combined with mask coefficients to get *m* instance $c_{m}(m\in \left\{1,2,\cdots ,M\right\}$. We can perform defect analysis on a single instance, removing interference from complex backgrounds.

### 2.2. Superpixel Segmentation

In the previous section, the image of each type of target equipment in the infrared image is segmented. In this section, the target equipment is detected individually. To make defect detection easier, we first perform superpixel segmentation. Superpixel segmentation forms superpixels from adjacent pixels in the image of target equipment with similar texture, color, luminance, or other characteristics. Thus, superpixels can be treated as processing units, reducing the complexity and time spent on the subsequent processing of the image [36]. Superpixel segmentation methods are generally classified into graph theory-based methods [37,38] and clustering-based methods [39,40,41]. Computation of cost functions in graph theory-based methods is complicated. In contrast, clustering-based methods has simple principles and good interpretability. The clustering-based simple linear iterative clustering (SLIC) algorithm obtains uniform compact superpixels, and it has good controllability and low operational complexity than other superpixel algorithms [42].

Inspired by the SLIC algorithm, the proposed model forms adjacent pixels with similar temperature characteristics *t* and spatial characteristics into superpixels $c_{m}^{n},n\in \left\{1,2,\ldots ,N\right\}$. Assume that there are *I* pixels in infrared image *c*, and the number of superpixels is *K*. Then the interval between the clustering centers $C_{k}$ is $S=\sqrt{I/K}$. The pixels 2S distance from the clustering center is iteratively clustered based on spatial similarity and temperature similarity, until the maximum number of iterations is reached. The formula for calculating the distance *D* between pixel *i* and the cluster center $C_{k}$ is as follows:

(1) $$D=\sqrt{{\left(\frac{d_{t}}{m_{t}}\right)}^{2}+{\left(\frac{d_{xy}}{m_{xy}}\right)}^{2}},$$

(2) $$d_{t}=\sqrt{{\left(t_{k}-t_{i}\right)}^{2}},$$

(3) $$d_{xy}=\sqrt{{\left(x_{k}-x_{i}\right)}^{2}+{\left(y_{k}-y_{i}\right)}^{2}},$$

where $d_{t}$ is the temperature distance between pixel *i* and the cluster center $C_{k}$, $d_{xy}$ is the spatial distance between pixel *i* and the cluster center $C_{k}$, $m_{t}$ and $m_{xy}$ are the maximum temperature distance and spatial distance obtained in the previous iteration, respectively.

Further, the superpixels $c_{m}^{n}$ of each instance are obtained, and the corresponding temperature characteristic $T_{m}^{n},n\in \left\{1,2,\ldots ,N\right\}$ is calculated by averaging the temperature of pixels in the superpixel. All temperature characteristics of $c_{m}^{n}$ lie between the maximum temperature $T_{m}^{\max}$ and the minimum temperature $T_{m}^{\min}$, i.e., $T_{m}^{n}\in \left[T_{m}^{\min},T_{m}^{\max}\right]$.

### 2.3. Defect Determination

**Figure 2:**
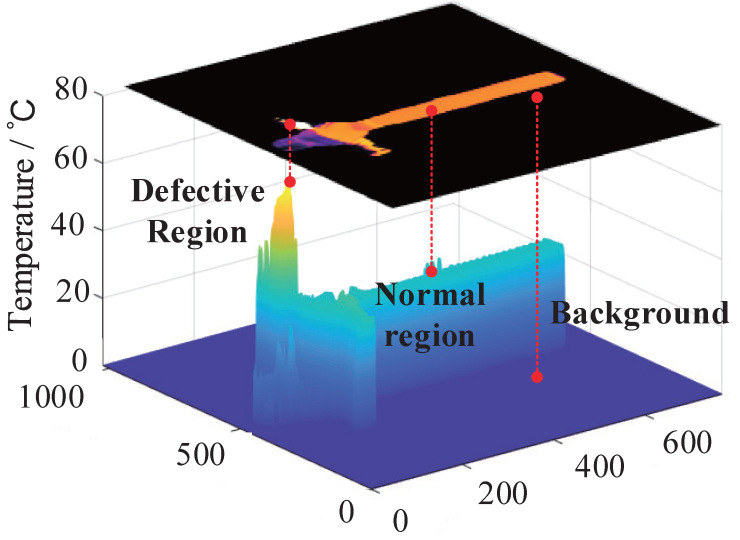
Infrared image of substation equipment and its temperature distribution.

After superpixel segmentation of the target equipment, we inspect the target equipment one by one to determine whether there exist defects. Figure 2 shows the target equipment of the background, normal region, and defective region with different temperature characteristics in the infrared image. The range of temperatures that the defect determination algorithm can identify is even broader than the temperatures range in Figure 2.

Different temperature characteristics correspond to different temperature probability densities. Thus, we can model the temperature probability density distribution of the instances to determine whether there are defects.

**Figure 3:**
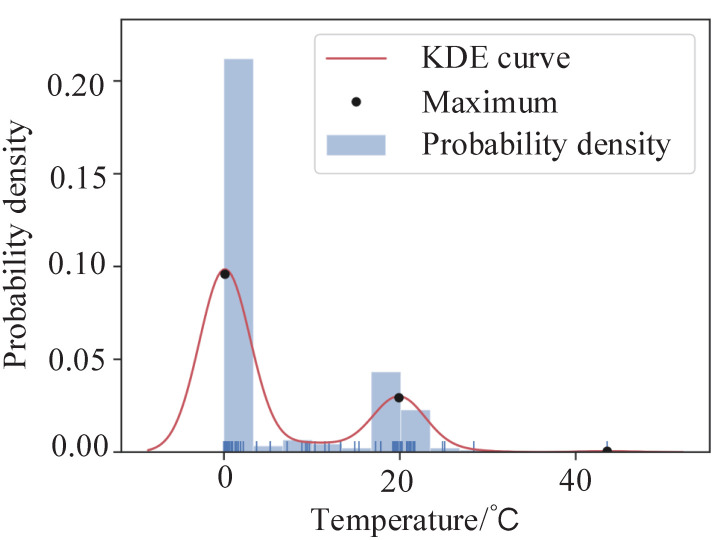
Temperature probability density distribution of $c_{m}$.

For instance $c_{m}$, the temperature probability density $T_{m}^{n}$ can be calculated by Equation (4), as shown by the blue histogram in Figure 3.

(4) $$f_{m}\left(n\right)=\frac{T_{m}^{n}}{\sum_{i=1}^{N}T_{m}^{i}},n\in \left[1,N\right].$$

However, the temperature probability density data are discretized, which cannot be used directly. Thus, we need to estimate the probability density function to approximate its specific distribution. The common probability density estimation methods include parametric probability density estimation and non-parameter probability density estimation. Kernel density estimation (KDE) [43] is a non-parameter probability density estimation method used to estimate the temperature probability density distribution of the data.

If there is a sufficiently small temperature region $A=[T_{m}^{A\min},T_{m}^{A\max}]$ with bandwidth *h*, the probability $P_{m}\left(A\right)$ of $T_{m}^{n}$ in *A* is

(5) $$P_{m}\left(A\right)=\int_{A}f_{m}\left(x\right)dx\approx f_{m}\left(x\right)\int_{T_{m}^{A\min}}^{T_{m}^{A\max}}dx=f_{m}\left(x\right)h.$$

Suppose the probability of *Z* out of *N* data falling into region *A* is

(6) $$P_{m}\left(A\right)=\frac{Z}{N}.$$

Then the temperature probability density becomes

(7) $$f_{m}\left(x\right)=\frac{Z}{Nh}.$$

The kernel density estimation of Equation 7 using the Gaussian kernel function obtains the temperature probability density function of instance $c_{m}$.

(8) $$f_{m}\left(x\right)=\frac{1}{Nh}\sum_{j=1}^{N}\frac{1}{\sqrt{2\pi }}e^{-\frac{1}{2}{\left(\frac{x-T_{m}^{j}}{h}\right)}^{2}}.$$

After that, the temperature probability density distribution function is visualized in Figure 3 by the red curve. The point of local maximum $O_{m}^{q}\left(\left(x_{m}^{q},y_{m}^{q_{}}\right),q=1,2,\ldots ,Q\right)$ is obtained, which is denoted by the black dots in Figure 3.

Based on the temperature probability density distribution function, we propose a determination strategy to find defects in infrared images. Due to the different temperature characteristics in the background, normal region, and defective region. Meanwhile, different temperature areas are shown in the temperature probability density distribution. Thus, the presence of $O_{m}^{q}$ and Q≥3 indicate the presence of a defect in the target equipment in this strategy. Then, through the application of the proposed algorithm, $x_{m}^{3}$ is used as the threshold, superpixels $c_{m}^{n}$ with temperature characteristics $T_{m}^{n}$ higher than $x_{m}^{3}$ are determined to be defective superpixels, automatically. Then, all adjacent defective superpixels are merged to determine the defective regions $D_{m}$ in instance $c_{m}$. Finally, all instances of the infrared image are traversed to obtain all the defective regions automatically. In addition, Algorithm 1 summarizes the whole programming procedure of the proposed TDDM.
**Algorithm 1** TDDM1:**Input:** Infrared image *c*, Number of superpixels *K*.2:**Output:** All defect regions in the infrared image.3:Obtain instance $c_{m}=\mathrm{Seg}\left(c\right),m=1,2,\ldots ,M$4:**for** m=1toM**do**5:   **for** n=1toN **do**6:     Compute superpixels $c_{m}^{n}$7:     Obtain temperature characteristic $T_{m}^{n}$8:   **end for**9:   Compute temperature probability density distribution $f_{m}$10:   Compute the local maximum $O_{m}^{q}\left(x_{m}^{q},y_{m}^{q}\right)$ of $f_{m}$, where q=1,2,…,Q11:   **if** Q≥3 **then**12:     **for** n=1toN **do**13:        **if** $T_{m}^{n}>x_{m}^{3}$ **then**14:          Determine $c_{m}^{n}$ defective15:        **else**16:          Determine $c_{m}^{n}$ normal17:        **end if**18:     **end for**19:     Merge all adjacent defective superpixels to obtain $D_{m}$20:   **else**21:     **Output:** No defect in the instance.22:   **end if**23:**end for**

## 3. Experiments

### 3.1. Data Preparation

**Figure 4:**
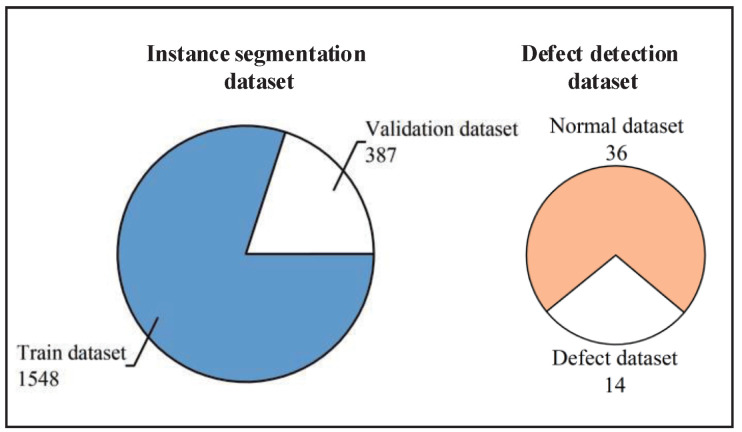
Dataset composition of the substation equipment infrared images.

The experimental infrared images in this article consist of five types of substation equipment, including insulator, current transformer, voltage transformer, bushing, and lightning arrester. The images were captured in a substation by the FLIR T600, where the infrared image resolution is 480 × 360 and the temperature resolution is 0.04 ℃. The dataset composition of the substation equipment infrared images in the experiments is illustrated in Figure 4. The instance segmentation dataset is used to train the instance segmentation module, in which the dataset all consists of the normal substation equipment images. The number of each type of equipment is shown in Table 2. In addition, the defect detection dataset is used to evaluate the performance of the TDDM.

### 3.2. Instance Segmentation Results and Analysis

The instance segmentation algorithm ran on Ubuntu 18.04LTS with NVIDIA 2080Ti. The training was conducted under the network framework PyTorch through Python3.8, accelerated by CUDA11.2. The current advanced instance segmentation algorithms, including SOLO, Mask R-CNN, MS R-CNN, and YOLACT, were compared experimentally. For training the instance segmentation algorithm, the batch size was set to 2, the SGD optimizer was used, the momentum value was 0.9, the initial learning rate was 0.001, and the number of training iterations was 60 epochs.

**Figure 5:**
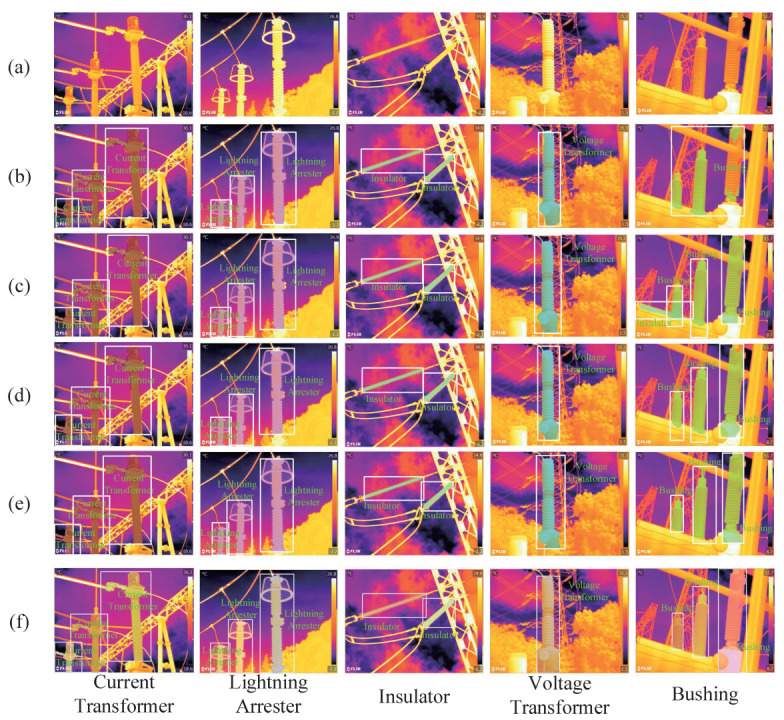
Comparison of segmentation results of different instance segmentation algorithms. (**a**) Original Images. (**b**) SOLO. (**c**) Mask R-CNN. (**d**) Mask Scoring R-CNN. (**e**) YOLACT. (**f**) Ground Truth.

To choose the optimal instance segmentation algorithm, a multi-target scene with a complex background was selected for testing. The performance indexes were mAP (mean average precision) and mAR (mean average recall), which are commonly used indexes in the current instance segmentation. SOLO, Mask R-CNN, Mask Scoring R-CNN, and YOLACT were tested on the instance segmentation dataset. The experiment results are shown in Figure 5 and Table 3.

In Table 3, YOLACT had the highest segmentation accuracies compared with the other three algorithms. The values are 67.0% and 74.0% in terms of the mAP and mAR metrics, which were 10.1% and 12.5% higher than the SOLO algorithms. As shown in Figure 5, Figure 5a are the original images and Figure 5f are the ground truth. The four algorithms are intuitively compared in Figure 5b–e, where the white rectangle represents the location of the substation equipment by the model. The pixels of instances belonging to the different categories are marked with different colors. It can be seen from Figure 5 that the YOLACT algorithm accurately located the substation equipment in infrared images and had typically higher quality masks. Thus, this study chose the YOLACT algorithm to segment substation equipment infrared images.

### 3.3. Compared with Other Superpixel Segmentation Methods

In this section, we compare SLIC [40] to several popular superpixel segmentation algorithms including Felzenszwalb [44], Quickshif [45], and Watershed [46] by the defect detection dataset. The performance of superpixel segmentation is quantitatively evaluated by two metrics, which are boundary recall (BR) and under-segmentation error (UE). BR is the most commonly used metric, which is the percentage of superpixels boundaries coinciding with ground truth boundaries.

(9) $$\mathrm{BR}=\frac{\mathrm{SP}}{\mathrm{GP}},$$

where SP is the number of segmentation results that meet the condition that should be the ground truth. GP is the total number of the segmentation result. The higher the BR denotes the better performance. UE is the ratio of calculated over-segmented superpixels. The more approaches zero of the UE, the superpixel approaches the ground truth. UE is defined as follow

(10) $$\mathrm{UE}=-1+\frac{1}{N}\sum {}_{\left|u_{m}\cap u_{n}\right|>\omega \left|u_{m}\right|}\left|u_{n}\right|,$$

where $u_{m}$ and $u_{n}$ are the pixel sets of the m-th superpixel and ground truth, respectively. ω is set to 0.05 for well established [47]. The lower the UE denotes the better performance.

**Figure 6:**
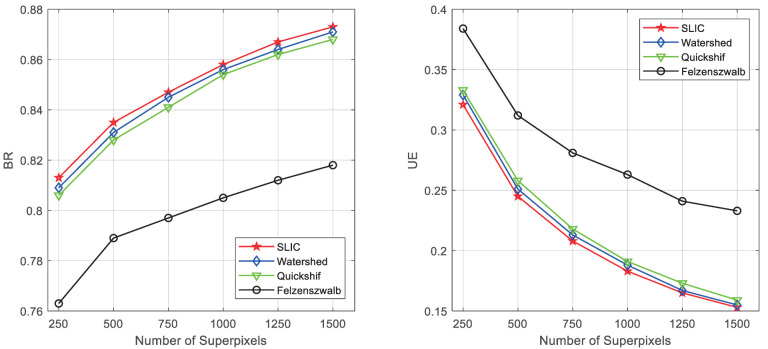
Comparison of superpixel segmentation algorithms performances.

As shown in Figure 6, it illustrates the comparative performance the methods on the defect detection dataset. The numbers of superpixels are set to 250, 500, 750, 1000, 1250, and 1500, respectively. From Figure 6, SLIC, Watershed, and Quickshif all obtain good performance since BR is higher than 0.86. The value of UE in SLIC is the lowest among all methods, this means that better compactness of superpixel segmentation can be achieved.

### 3.4. Defect Detection Results and Analysis

We adopted four widely used metrics for the quantitative evaluations of defect detection performance: precision ($P_{r}$), recall ($R_{e}$), $F_{1}$, and mean running time (mRN). A higher evaluation value indicates better performance, calculated as follows.

(11) $$P_{r}=\frac{\mathrm{TP}}{\mathrm{TP}+\mathrm{FP}},$$

(12) $$R_{e}=\frac{\mathrm{TP}}{\mathrm{TP}+\mathrm{FN}},$$

(13) $$F_{1}=\frac{2*P_{r}*R_{e}}{P_{r}+R_{e}},$$

where TP and denote the number of correctly detected defects. TP+FP and TP+FN denote the total number of detected defects and the total number of actual defects, respectively. $F_{1}$ is the harmonic mean of $P_{r}$ and $R_{e}$.

We use mean intersection over union (mIoU) to calculate the accuracy of defect region localization. The mIoU is defined as

(14) $$\mathrm{mIoU}(G_{T},P_{M})=\sum_{m=1}^{M}\frac{\mathrm{Area}(G_{T}^{m}\cap P_{D}^{m})}{\mathrm{Area}(G_{T}^{m}\cup P_{D}^{m})},$$

where $G_{T}^{m}$ is the ground truth and $P_{D}^{m}$ is the predicted region.

**Figure 7:**
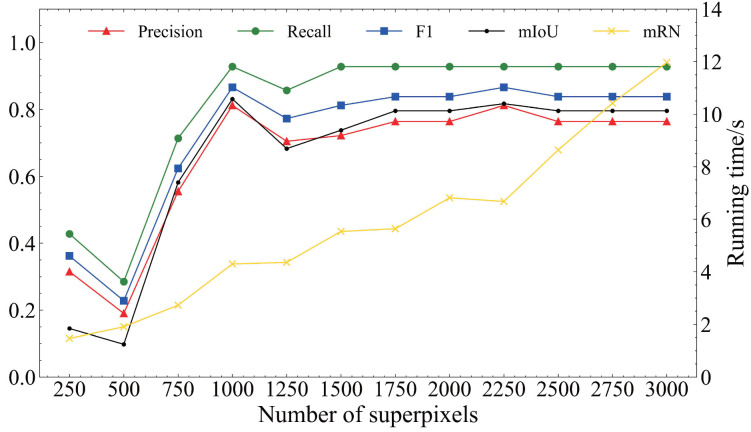
Results of ablation experiments on the number of superpixels.

To verify the effectiveness of the TDDM, the defect detection datasets are input to the TDDM. To choose the best parameter of the number of superpixels *K*, we set *K* from 250 to 3000 with an interval of 250 for the ablation experiments. When K=1000, TDDM has achieved the best defect detection performance. The values of precision, recall, $F_{1}$, and mIoU were 0.812, 0.928, 0.866 and 0.831. When K=2250, the model had acceptable precision and recall values performance, but the model running time became longer. Moreover, the running time of TDDM increased with *K*. Thus, in a word, the selection of an appropriate *K* is important. Table 4 and Figure 7 show the comparison with a different number of superpixels *K* to the defect detection dataset.

To evaluate the superiority of the proposed method, some ablation experiments were performed on TDDM. (1) Evaluate the advantage of the superpixel segmentation algorithm (SSA) as a preprocessing for defect detection. (2) Evaluate the advantage of the DCNN + superpixel method for defect detection. Table 5 lists the results of the ablation experiment. As shown in Table 5, the SSA can provide more details and reduce the complexity of the subsequent detection determination. When the objects are extracted firstly by DCNN, the metrics for evaluating accuracy have improved. It indicates that DCNN can overcome the problem of complex background in infrared images. The model achieved superior results when both DCNN and SSA were used. $P_{r}$, $R_{e}$, $F_{1}$, mIoU are reached 0.812, 0.928, 0.866, and 0.831, respectively, which were the highest values.

**Figure 8:**
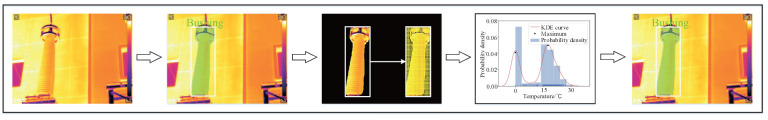
Process of the normal bushing infrared image detection.

**Figure 9:**
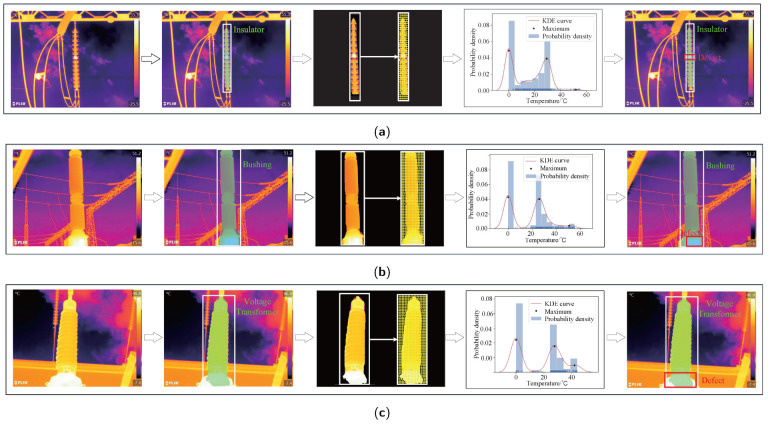

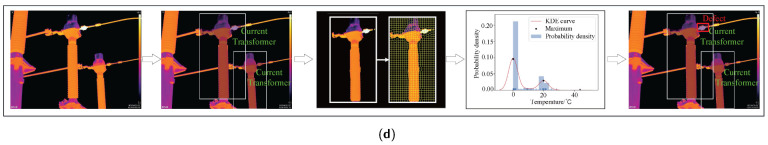
Process of the defect infrared image detection. (**a**) Insulator. (**b**) Bushing. (**c**) Voltage Transformer. (**d**) Current Transformer.

As shown in Figure 8 and Figure 9, the intuitive defect detection process of the TDDM in this paper is on the defect detection dataset.In the intuitive experiment results, the different categories have displayed.

Figure 8 shows the process of the normal bushing infrared image detection. In the fourth column, the temperature probability density distribution of the bushing has only two local maxima, which reflects that the substation equipment is no defect. This demonstrates that the TDDM is effectively applied in detecting normal substation equipment.

Figure 9 shows the entire detection flow of the TDDM to the defect-located infrared images. From left to right are the input infrared images, instance segmentation, superpixel segmentation, defect determination, and defect detection results. At the penultimate column, there are three maxima in the temperature probability density distribution of target equipment, representing the equipment exist defect. The target equipment defect detection results are shown in the last column. The white rectangle denotes the target equipment, and the red rectangle represents the location of the defective regions. As can be seen that, TDDM accurately located the defect in substation equipment against a complex background.

## 4. Discussion

In this paper, a two-level model is proposed for the problem of defect detection in substation equipment infrared images. On the basis of extracting substation equipment in the complex background through instance segmentation and superpixel segmentation methods, and realizing defect detection of substation equipment through temperature probability density distribution calculation and adaptive defect detection strategy. Compared with the traditional manual inspection, the proposed method can reduce the resources of labor and material; compared with the end-to-end deep learning method, the presented method in this paper does not require many defect datasets. The operating status of the substation equipment is closely relevant to the stability of the power system, which makes the defects detection of the substation equipment significant.

In the future, our research will not be limited to the substation equipment in this paper and will be applied to other electrical equipment. In fact, according to the characteristic of infrared thermal imaging, the majority of electrical equipment infrared images will show a certain temperature probability density distribution, which is the physical characteristic. The proposed method is based on this characteristic to detect defects precisely. Thus, based on this physical characteristic, we believe the method will be applicable to other cases where may occur defects in electric power, such as medical equipment, airplanes, and industrial equipment.

## 5. Conclusions

This study proposes a novel defect detection model named TDDM for infrared images of substation equipment. Considering the defective substation equipment infrared images are difficult to acquire, and the data-driven end-to-end model cannot be trained. Thus, the two-level defect detection method is presented. In the proposed TDDM, we take advantage of the fact that the instance segmentation has superior performance to extract the target in the redundant background. Meanwhile, the part of defect detection of TDDM is unsupervised and is not limited by the dataset. Furthermore, we evaluated the proposed model on the defect detection dataset, which accurately detects defects of substation equipment in infrared images. In the future, we would like to combine the RGB information to improve substation inspection tasks. In addition, the technology will be applied to more substation equipment.

## Figures and Tables

**Table 1:** The description of specific backbone configuration parameters.

Layer Name	Structure	Convolution Kernel	Feature Map Size
Input Layer	-	-	640 × 640
Conv1	-	7 × 7 × 64, stride 2	320 × 320
Pool1	Maxpool	3 × 3 × 64, stride 2	160 × 160
Conv2_x	Bottleneck	$\left[\begin{array}{c} 1\times 1\times 64\\ 3\times 3\times 64\\ 1\times 1\times 256 \end{array}\right]\times 3$	160 × 160
Conv3_x	Bottleneck	$\left[\begin{array}{c} 1\times 1\times 128\\ 3\times 3\times 128\\ 1\times 1\times 512 \end{array}\right]\times 4$	80 × 80
Conv4_x	Bottleneck	$\left[\begin{array}{c} 1\times 1\times 256\\ 3\times 3\times 256\\ 1\times 1\times 1024 \end{array}\right]\times 6$	40 × 40
Conv5_x	Bottleneck	$\left[\begin{array}{c} 1\times 1\times 512\\ 3\times 3\times 512\\ 1\times 1\times 2048 \end{array}\right]\times 3$	20 × 20

**Table 2:** Number of each type of equipment in the instance segmentation dataset.

Equipment	Number
Insulator	919
Current transformer	413
Lightning arrester	289
Bushing	161
Voltage transformer	153

**Table 3:** Comparison of instance segmentation algorithms.

Method	mAP/%	mAR/%
SOLO	56.9	61.5
Mask R-CNN	63.6	70.4
Mask Scoring R-CNN	65.1	70.9
YOLACT	67.0	74.0

**Table 4:** Detection performance for different numbers of *K*.

Number of *K*	$P_{r}$	$R_{e}$	$F_{1}$	mIoU	mRN
250	0.315	0.428	0.362	0.145	1.47
500	0.190	0.285	0.228	0.098	1.91
750	0.555	0.714	0.624	0.582	2.73
1000	0.812	0.928	0.866	0.831	4.30
1250	0.705	0.857	0.773	0.683	4.36
1500	0.722	0.928	0.812	0.738	5.54
1750	0.764	0.928	0.838	0.796	5.64
2000	0.764	0.928	0.838	0.796	6.82
2250	0.812	0.928	0.866	0.817	6.68
2500	0.764	0.928	0.838	0.796	8.64
2750	0.764	0.928	0.838	0.796	10.42
3000	0.764	0.928	0.838	0.796	11.97

**Table 5:** Ablation experiment of TDDM.

DCNN	SSA	$P_{r}$	$R_{e}$	$F_{1}$	mIoU	mRN
✓		0.764	0.928	0.838	0.796	21.34
	✓	0.555	0.714	0.624	0.582	3.62
✓	✓	0.812	0.928	0.866	0.831	4.30

## Data Availability

Not applicable.

## Abbreviations

The following abbreviations are used in this manuscript:

DCNN	Deep Convolutional Neural Network
TDDM	Two-level Defect Detection Model
UHF	Ultra-high Frequency
FDR	frequency Domain Reflectometry
SNR	Signal-noise-ratio
TLD	Transmission Line Detection
NMS	Non-maximum Suppression
SLIC	Simple Linear Iterative Clustering
KDE	Kernel Density Estimation
mAP	Mean Aaverage precision
mAR	Mean Aaverage Recall
mRN	Mean Running Time
SSA	Superpixel Segmentation Algorithm
TP	True Positive
FP	False Positive
FN	False Negative
